# PI-RADS v2.1 Combined With Prostate-Specific Antigen Density for Detection of Prostate Cancer in Peripheral Zone

**DOI:** 10.3389/fonc.2022.861928

**Published:** 2022-04-08

**Authors:** Jing Wen, Tingting Tang, Yugang Ji, Yilan Zhang

**Affiliations:** ^1^Department of Medical Imaging, Jiangsu Vocational College of Medicine, Yancheng, China; ^2^Department of Radiology, Yancheng First Peoples’ Hospital, Yancheng, China

**Keywords:** mpMRI, prostate neoplasm, diagnostic performance, PSAD; PI-RADS

## Abstract

**Purpose:**

To evaluate the diagnostic performance of combining the Prostate Imaging Reporting and Data System (PI-RADS) scoring system v2.1 with prostate-specific antigen density (PSAD) to detect prostate cancer (PCa).

**Methods:**

A total of 266 participants with suspicion of PCa underwent multiparametric magnetic resonance imaging (mpMRI) in our hospital, after at least 4 weeks all patients underwent subsequent systematic transrectal ultrasound (TRUS)-guided biopsy or MRI-TRUS fusion targeted biopsy. All mpMRI images were scored in accordance with the PI-RADS v2.1, and univariate and multivariate logistic regression analyses were performed to determine significant predictors of PCa.

**Results:**

A total of 119 patients were diagnosed with PCa in the biopsy, of them 101 patients were diagnosed with clinically significant PCa. The multivariate analysis revealed that PI-RADS v2.1 and PSAD were independent predictors for PCa. For peripheral zone (PZ), the area under the ROC curve (AUC) for the combination of PI-RADS score and PSAD was 0.90 (95% CI 0.83-0.96), which is significantly superior to using PI-RADS score (0.85, 95% CI 0.78-0.93, P=0.031) and PSAD alone (0.83, 95% CI 0.75-0.90, P=0.037). For transition zone (TZ), however, the combination model was not significantly superior to PI-RADS alone, with AUC of 0.94 (95% CI 0.89-0.99) vs. 0.93 (95% CI 0.88-0.97, P=0.186).

**Conclusion:**

The combination of PI-RADS v2.1 with PSAD could significantly improve the diagnostic performance of PCa in PZ. Nevertheless, no significant improvement was observed regarding PCa in TZ.

## Introduction

PCa is the most common malignancy among males in Northern America and Europe, where one in nine men will be diagnosed with prostate cancer at some point during their lifetime (1, 2). Compared with conventional examinations such as serum prostate-specific antigen (PSA) and digital rectal examination (DRE) (3, 4), mpMRI has demonstrated more accuracy in localizing, diagnosis, and staging of PCa. Previous studies showed MRI-targeted fusion biopsy is superior to the conventional standard transrectal ultrasonography (TRUS)–guided biopsy (5–8). Besides, a rencently published study demonstrated that MRI-targeted fusion biopsy could significantly reduce the risk of Gleason Score (GS) 3 + 4 upgrading at radical prostatectomy compared to standard biopsy (9). In 2019, the American College of Radiology (ACR) and the European Society of Urogenital Radiology (ESUR) updated the Prostate Imaging-Reporting and Data System (PI-RADS) to version 2.1, which is a standardized scoring system for performing, interpreting, and reporting the PCa with mpMRI (10–12). Despite this guideline having been widely applied in clinical practice, the inter-reader agreement is not very high and the reported diagnostic performance varied widely (13). Furthermore, using PI-RADS alone may result in a moderate diagnostic accuracy for PCa (14), a recent study revealed that the pooled sensitivity and specificity for version 2.1 were 0.87 and 0.74, respectively (15). Therefore, a combination of MRI with other clinical parameters and biomarkers should be considered to improve the diagnostic performance. Among several potential factors, PSAD was considered as a promising predictor for the presence of PCa (16–18).

The National Comprehensive Cancer Network (NCCN) guidelines suggest a PSAD value below 0.15 ng/ml/ml for very low-risk cancer (19), and several studies have demonstrated that PSAD could be regarded as an independent predictor or in conjunction with other clinical information for staging or evaluation of PCa (16, 18, 20, 21). Thus, the objective of our study was to evaluate whether the diagnostic performance of PI-RADS v2.1 could be improved by adding PSAD.

## Methods and Materials

### Study Population

**Figure 1:**
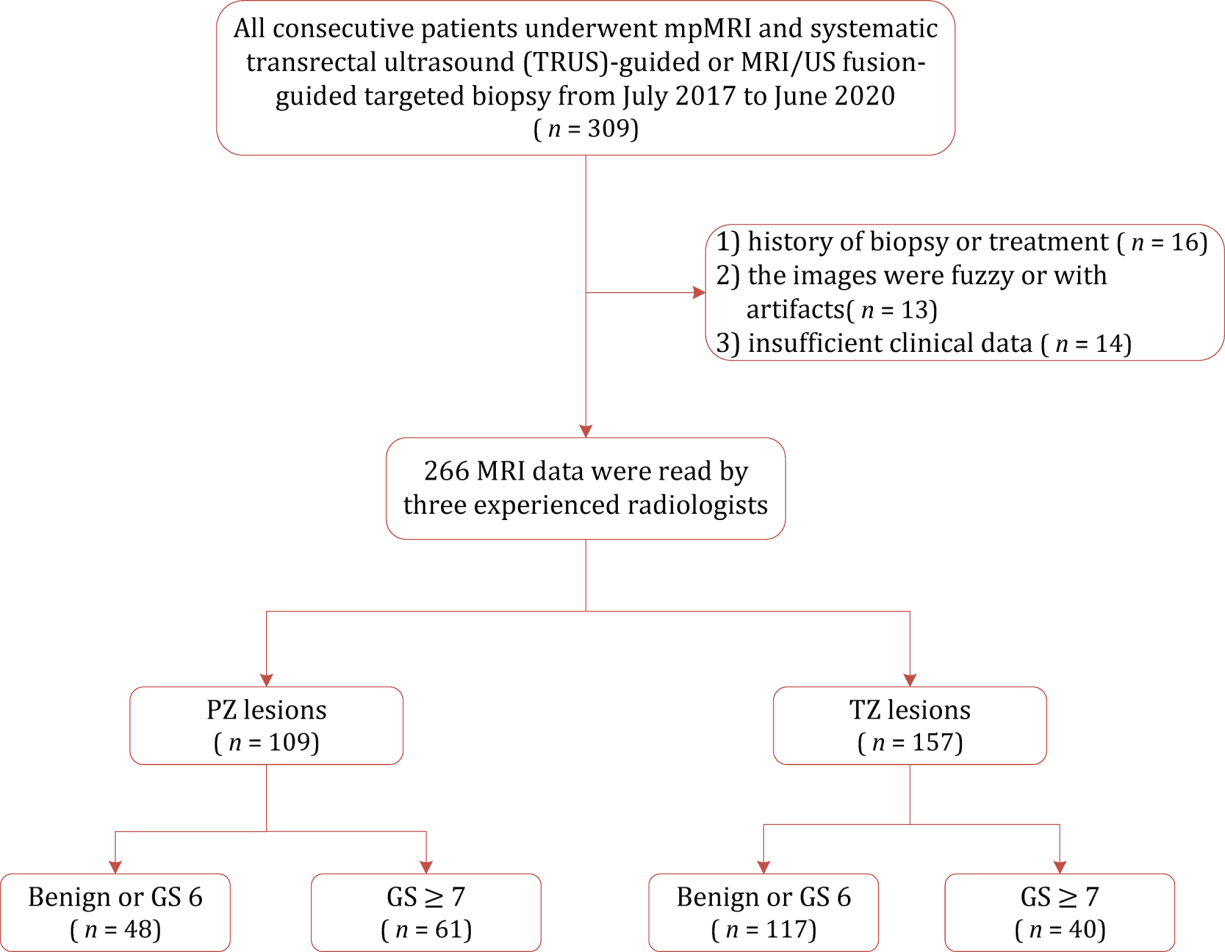
Flowchart of the study population with the exclusion criteria. GS, gleason score; PZ, peripheral zone; TZ, transition zone.

This retrospective study was approved by our institutional review board who waived the requirement for informed consent and was conducted in accordance with the Declaration of Helsinki. We searched the electronic database of our institution for consecutive 309 patients who underwent mpMRI and subsequent systematic TRUS-guided prostate biopsy and/or MRI-TRUS fusion targeted biopsy between July 2017 and June 2020. We excluded 43 patients for reasons as follows: 1) history of biopsy or treatment; 2) the images were fuzzy or with artifacts; and 3) missing clinical data. The patient selection process is described in **Figure 1**.

### Image Acquisition

All mpMRI examinations were performed on a 3.0 T MRI scanner (Philips Ingenia, The Netherlands) before biopsy, with a 32-channel body phased-array coil. The imaging acquisition protocol was in compliance with the PI-RADS v2.1 criteria, which includes high-resolution axial and sagittal T2-weighted imaging (T2WI), and axial diffusion-weighted imaging (DWI). The DWI sequences were obtained with multiple ***b*** values (***b*** =0, 100, 1,000, 2,000 s/mm^2^), in which the values of 100 and 2000 s/mm^2^ were used to visually evaluate and analyze the apparent diffusion coefficient (ADC) map.

### Image Analysis

All examinations were independently reviewed by two fellowship-trained radiologists (***W.J.***, with 8 years of experience and ***T.T.T.***, with 3 years of experience) in prostate cancer imaging, who were blinded to clinical information and pathologic findings. The PI-RADS v2.1 guidelines were used to score each lesion based on the DWI and T2WI sequences, and the highest overall PI-RADS score of each mpMRI scan was used.

### Prostate Biopsy

All patients underwent a 10-core systematic TRUS-guided biopsy after at least 4 weeks of the MRI examination, and MRI-TRUS fusion targeted biopsy was performed for lesions with PI-RADS 2.1 score ≥3. The MRI-TRUS fusion targeted biopsy was performed with ESAOTE Mylab Twice color Doppler ultrasound device, which was equipped with real-time virtual sonography (RVS) imaging fusion system. The prostate biopsies were performed by a qualified urologist who with experience of at least 200 MRI-TRUS fusion biopsies.

### Pathology

All specimens were assessed by an experienced pathologist (with 6 years of experience) in our institution according to the International Society of Urological Pathology (ISUP) 2014 updated Gleason score grading system (22, 23). The PSAD was calculated by serum total PSA divided by the prostate volume, which was estimated according to PI-RADS v2.1 recommends that the maximum anteroposterior diameter and longitudinal diameters measured on midsagittal T2WI, while the maximum transverse diameter measured on the axial T2WI.

### Statistical Analysis

All statistical analyses were conducted by using STATA 16.0, and a *P* value of less than 0.05 was considered statistically significant. The inter-reader agreement of the PI-RADS v2.1 score was evaluated by weighted Cohen’s kappa (*κ*) statistic: a *κ* value of <0.20 indicates slight agreement, a *κ* value between 0.21 and 0.40, fair agreement, a *κ* value between 0.41 and 0.60, moderate agreement, a *κ* value between 0.61 and 0.80, substantial agreement, and a *κ* value of between 0.81 and 1.00, almost perfect agreement.

We performed univariable logistic regression analysis for each variable to investigate the significant predictors of PCa, which included age, PSA level, MRI prostate volume, PSAD, and PI-RADS v2.1 score. Afterward, multivariable binary logistic regression analysis was performed to explore the significant clinical factors for PCa. The AUC was calculated and used to determine the diagnostic performance of variables, and the best combination was defined as the one with the largest AUC. The diagnostic sensitivity and specificity with their 95% confidence intervals (95% CIs) were calculated. A nomogram for the best combination in the multiple logistic regression analyses was generated using “nomology” command in STATA 16.0.

## Results

### Patient Characteristics

The patient characteristics are summarized in **Table 1**. The median age of 266 patients included was 71.3 years, with a median PSA level of 11.33 (interquartile range [IQR] 6.85-21.4) and median PSAD of 0.21 (IQR 0.12-0.49). A total of 119 patients (39.3%) were diagnosed with PCa in the biopsy, of whom 101 patients were diagnosed with clinically significant PCa (GS ≥ 7 or tumor size ≥ 0.5 mL), and the remaining 18 patients were diagnosed with clinically insignificant prostate cancer (GS=3+3).

**Table 1:** Patient characteristics. Clinical Characteristics of Patients Analyzed in This Study.

Characteristics	Value
Patients (n=266)	
Age (year, mean±SD)	71.34±8.23
PSA (ng/ml, IQR)	11.33 (6.85-21.4)
PSAD (ng/ml/ml, IQR)	0.21 (0.12-0.49)
Volume (ml, IQR)	52 (36.22-72.38)
Gleason score	
3+3	18
3+4	19
4+3	19
4+4	27
4+5	12
5+4	11
5+5	13
Location	
PZ	109
TZ	157

IQR, interquartile range; PSA, prostate-specific antigen; PSAD, prostate-specific antigen density; SD, standard deviation.

### Diagnostic Performance of PI-RADS v2.1

The sensitivity and specificity of PI-RADS v2.1 category ≥3 for diagnosing PCa of the whole gland were 96.2% (95% CI 90.5%-98.5%) and 61.3% (95% CI 52.5%-69.4%), respectively. For 157 lesions located in the TZ, a cutoff threshold ≥3 yielded a sensitivity of 94.4% (95% CI 84.9%-98.1%) and specificity 69.9% (95% CI 60.5%-77.9%). Regarding 109 lesions located in the PZ, a cutoff threshold ≥3 yielded a slightly higher sensitivity (98.7%, 95% CI 93.0%-99.8%) but significantly lower specificity (18.6%, 95% CI 8.9%-35.3%). When used PI-RADS category ≥4 as the cutoff threshold, the sensitivity and specificity for diagnosing PCa of the whole gland were 89.4% (95% CI 82.0%-94.0%) and 84.7% (95% CI 77.3%-90.0%), respectively. Regarding TZ, this cutoff yielded a sensitivity of 90.7% (95% CI 80.1%-96.0%) and 89.3% (95% CI 81.9%-93.9%). As for PZ, a cutoff threshold ≥4 yielded slightly higher sensitivity (92.2%, 95% CI 84.0%-96.4%) but lower specificity (65.6%, 95% CI 48.3%-79.6%). The weighted *κ* value of 0.52 (95% CI 0.50-0.56) suggested that the inter-observer agreement was moderate for PI-RADS v2.1. **Table 2** shows the detailed diagnostic accuracy.

**Table 2:** Diagnostic accuracy of PI-RADS and PSAD.

Cutoff	Zonal	Sensitivity	95% CI	Specificity	95% CI
PI-RADS 2.1 ≥ 3	PZ	98.7%	93.0%-99.8%	18.6%	8.9%-35.3%
	TZ	94.4%	84.9%-98.1%	69.9%	60.5%-77.9%
PI-RADS 2.1 ≥ 4	PZ	92.2%	84.0%-96.4%	65.6%	48.3%-79.6%
	TZ	90.7%	80.1%-96.0%	89.3%	81.9%-93.9%
PASD ≥ 0.15 ng/ml/ml	PZ	90.9%	82.2%-96.3%	53.1%	34.7%-70.9%
	TZ	96.3%	87.3%-99.5%	53.4%	43.3%-63.3%
Optimal PSAD (ng/ml/ml)	PZ (0.25)	72.7%	61.4%-82.3%	81.3%	63.6%-92.8%
	TZ (0.33)	77.8%	64.4%-88.0%	86.4%	78.2%-92.4%

CI, confidence interval; PSA, prostate-specific antigen; PSAD, prostate-specific antigen density; PZ, peripheral zone; TZ, transition zone.

Concerning PSAD, a cutoff value ≥0.15 ng/mL/mL yielded sensitivity of 96.3% (95% CI 87.3%-99.5%), with specificity of 53.4% (95% CI 43.3%-63.3%) in TZ. Whereas for PZ, the generated sensitivity and specificity for this cutoff threshold were 90.9% (95% CI 82.2%-96.3%) and 53.1% (95% CI 34.7%-70.9%). The optimal cutoff using PSAD for TZ was ≥0.33 ng/mL/mL, at which the sensitivity and specificity were 77.8% (95% CI 64.4%-88.0%) and 86.4% (95% CI 78.2%-92.4%). The optimal cutoff using PSAD for PZ was ≥0.25 ng/mL/mL, at which the sensitivity and specificity were 72.7% (95% CI 61.4%-82.3%) and 81.3% (95% CI 63.6%-92.8%).

### Logistic Regression Analyses of PCa

The univariate logistic regression analysis revealed that the variables of PSA, prostate volume, PSAD, and PI-RADS were significant independent predictors for PCa. However, PSA and prostate volume were excluded because they were strongly correlated with PSAD. Eventually, only PSAD and PI-RADS score were included in the multivariable logistic regression analyses. **Table 3** shows the details of logistic regression analyses.

**Table 3:** Univariate and multivariate logistic regression analyses. Logistic Regression Analysis.

Variable	β Coefficient	Odd Ratio	95% CI	*P* Value
Univariable logistic regression model for PZ				
Volume	-0.004	1.0	0.98-1.01	0.517
PSAD	3.63	37.66	3.3-429.1	0.002
PI-RADS	1.80	6.05	3.1-11.9	<0.001
Age	0.09	1.09	1.03-1.16	0.003
PSA	0.06	1.06	1.01-1.1	0.014
Univariable logistic regression model for TZ				
Volume	-0.3	0.97	0.96-0.99	<0.001
PSAD	2.68	14.57	4.64-45.76	<0.001
PI-RADS	1.92	6.82	4.05-11.49	<0.001
Age	0.02	1.02	0.98-1.06	0.46
PSA	0.03	1.03	1.02-1.05	<0.001
Multivariable logistic regression model for PZ				
PSAD	2.33	10.3	1.01-105.4	0.006
PI-RADS	1.56	4.78	2.26-10.09	<0.001
Multivariable logistic regression model for TZ				
PSAD	1.39	4.03	1.13-14.36	0.032
PI-RADS	1.75	5.76	3.64-9.12	<0.001

PI-RADS, Prostate Imaging Reporting and Data System version 2.1; PSA, prostate-specific antigen; PSAD, prostate-specific antigen density; PZ, peripheral zone; TZ, transition zone.

**Figure 2:**
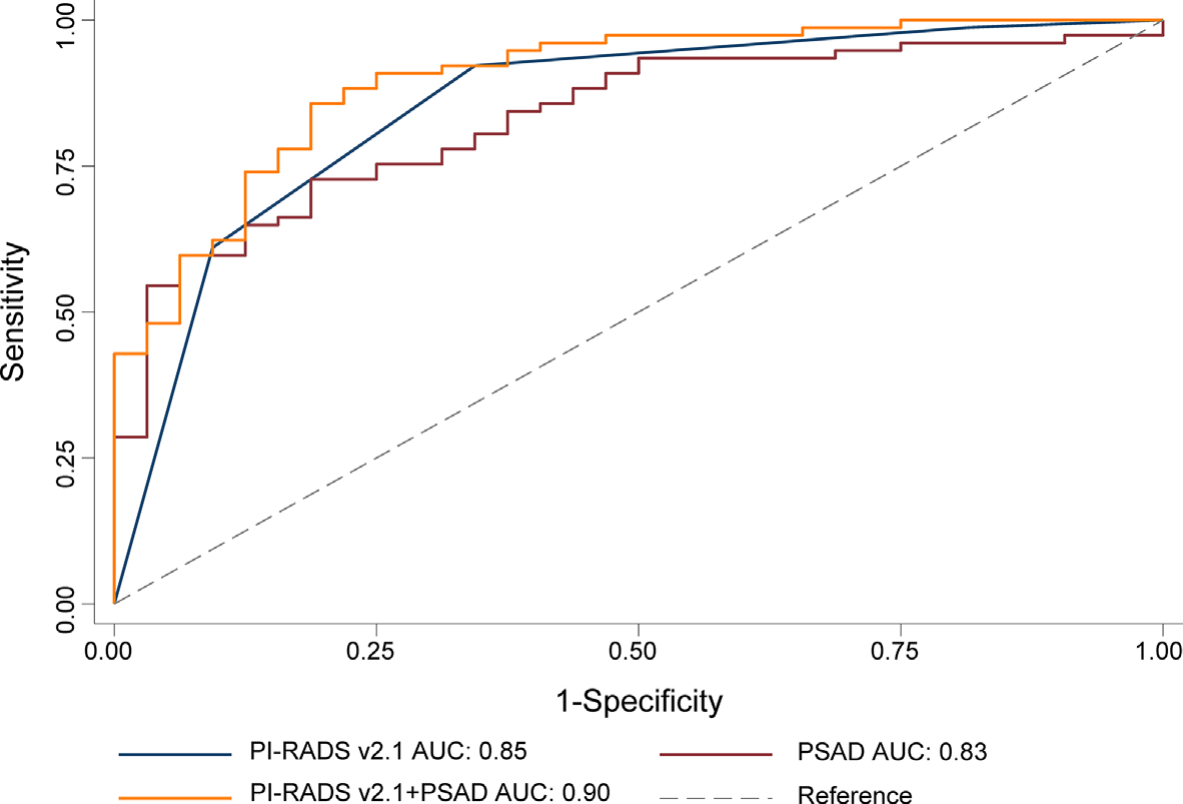
ROC for the comparison of PI-RADS+PSAD with PI-RADS and PSAD alone for the diagnosis of the prostate cancer. PI-RADS, Prostate Imaging Reporting and Data System version 2.1; PSAD, prostate-specific antigen density; AUC, area under the ROC curve.

**Figure 3:**
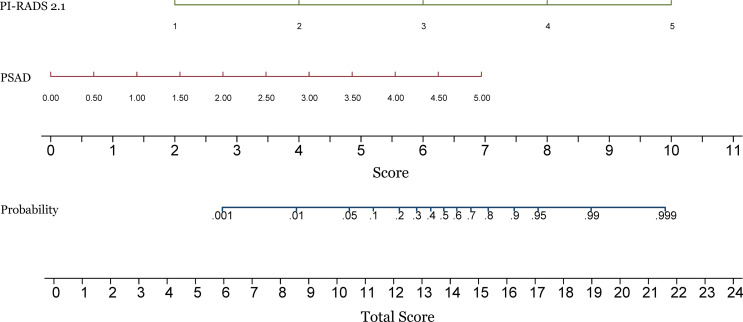
Construction of a nomogram for predicting the probability of prostate cancer in the peripheral zone. PI-RADS, Prostate Imaging Reporting and Data System version 2.1; PSAD, prostate-specific antigen density.

For the whole gland, the predictive power of the combination of PI-RADS and PSAD (AUC 0.94, 95% CI 0.91-0.97) was significantly superior to each of them alone (AUC 0.92, 95% CI 0.88-0.95, P=0.018, and 0.83, 95% CI 0.77-0.88, P<0.001, respectively). We performed analyses according to the location of the lesions. Regarding PZ, PI-RADS in conjunction with PSAD yielded AUC of 0.90 (95% CI 0.83-0.96), which is substantially superior to PI-RADS (AUC 0.85, 95% CI 0.78-0.93, P=0.037) and PSAD (AUC 0.83, 95% CI 0.75-0.90, P=0.031) alone, which is demonstrated in **Figure 2**. As for TZ, however, the improvement of combination (AUC 0.94, 95% CI 0.89-0.99) was not significant as compared to PI-RADS (AUC 0.93, 95% CI 0.88-0.97, P=0.186), but substantially better than PSAD (AUC 0.88, 95% CI 0.82-0.94, P=0.007). The detailed AUC analyses are presented in **Table 4**. A nomogram was generated for predicting PZ PCa, which is based on the combination of PI-RADS score and PSAD (**Figure 3**).

**Table 4:** ROC curve analysis for predicting prostate cancer.

Variable	AUC (95% CI)	P Value
Whole Gland		
PSAD	0.83 (0.77-0.88)	<0.001
PI-RADS	0.92 (0.88-0.95)	0.018
PI-RADS+PSAD	0.94 (0.91-0.97)	–
PZ		
PSAD	0.83 (0.75-0.90)	0.037
PI-RADS	0.85 (0.78-0.93)	0.031
PI-RADS+PSAD	0.90 (0.83-0.96)	–
TZ		
PSAD	0.88 (0.82-0.94)	0.007
PI-RADS	0.93 (0.88-0.97)	0.186
PI-RADS+PSAD	0.94 (0.89-0.99)	–

AUC, area under the ROC curve; CI, confidence interval; PI-RADS, Prostate Imaging Reporting and Data System version 2.1; PSA, prostate-specific antigen; PSAD, prostate-specific antigen density; PZ, peripheral zone; TZ, transition zone.

## Discussion

Our study demonstrated that both PI-RADS v2.1 and PSAD had a high diagnostic performance for the detection of PCa. The optimal cutoff threshold of PI-RADS score for both PZ and TZ was ≥4, at which the sensitivities were 92.2% and 90.7%, with specificities of 65.6% and 89.3%, respectively. The AUC of PI-RADS v2.1 score for TZ and PZ were 0.85 and 0.93, respectively. According to our analyses, the cutoff threshold of PSAD ≥0.15 ng/mL/mL yielded high sensitivity (90.9% and 96.3% for PZ and TZ) but low specificity (53.1% and 53.4%), with corresponding AUC were 0.83 and 0.88 for PZ and TZ. While in conjunction of PI-RADS score with PSAD, we noted that the diagnostic performance was superior to using these two predictors alone, especially for PZ lesions. The AUC for the combination was 0.94, compared with 0.92 for PI-RADS (P=0.018) and 0.83 for PSAD (P<0.001) alone. We performed analyses according to zonal location, and the combination of AUC 0.90 suggested that the diagnostic accuracy was significantly improved in PZ, where AUC for PI-RADS and PSAD were 0.85 (P=0.031) and 0.83 (P=0.037), respectively. In TZ, however, no significant improvement in diagnostic performance was observed while adding PSAD to PI-RADS, with AUC improved from 0.93 to 0.94 (P=0.186), but it was significantly superior to using PSAD as an independent predictor (AUC 0.88, P=0.007).

Several previous studies have demonstrated that the diagnostic performance was significantly improved as the combination of PI-RADS and PSAD (16, 20, 24). To our knowledge, however, there was no published study on the combination of PI-RADS v2.1 with PSAD for PCa PZ lesions. Distler et al. demonstrated that the NPV of PI-RADS can be improved by including PSAD, with an AUC of 0.79 (95% CI 0.76-0.82) (24). Another study performed by Washino et al. showed that patients with a PI-RADS v2 score of ≤3 and PSA density of <0.15 ng/mL/mL may avoid unnecessary biopsies (16). In our study, however, the optimal cutoff threshold was slightly higher, with 0.25-0.33 ng/mL/mL. Our study demonstrated that the combination of PSAD and PI-RADS was benefitted for detection of any PCa in PZ. In several recent studies, Roscigno et al. demonstrated that that mpMRI is not accurate enough during the AS follow-up, and it is still necessary to combine mpMRI with other clinical variables to improve the predictive accuracy (25, 26).

As a standardized reporting system, the PI-RADS has been validated and widely applied in clinical practice. Two meta-analyses demonstrated that the pooled sensitivity for PI-RADS v1 and v2 were 0.78 and 0.89, with the specificity of 0.79 and 0.73, respectively (27, 28). A more recent study including 14 head-to-head comparisons showed that PI-RADS v2 has slightly higher sensitivity but at the expense of minor decreased specificity (29). To address the problem of variability across institutions and readers, especially for lesions in the transition zone, the ESUR updated PI-RADS to v2.1 in 2019 (12). However, a study revealed that there was no significant difference in diagnostic performance between PI-RADS v2 and v2.1 (15).

Although PI-RADS v2.1 demonstrated good overall performance for the diagnosis of PCa, the specificity for PZ is still lower and thus leads to unnecessary biopsy. Moreover, the sensitivity may vary widely and depend on radiologists’ own experience (30–32). As a promising predictor, PSAD has shown promising potential for the detection of PCa. However, using PSAD as independent predictor alone results in lower diagnostic performance, moreover, the cutoff value varied widely (21, 33). A prior study showed that with a cutoff of 0.15 ng/ml/ml the sensitivity and specificity for csPCa were 0.70 and 0.70, respectively. In that study, the highest Youden’s index was at PSAD of 0.20 ng/ml/ml, which yielded a sensitivity of 0.70 and specificity of 0.79. According to our results, however, the optimal cutoff thresholds for distinguishing PCa were 0.25 ng/ml/ml for PZ and 0.33 ng/ml/ml for TZ. Therefore, the PSAD should be employed with other methods for the detection of PCa in clinical practice. In summary, the combination of PI-RADS v2.1 score and PSAD could be helpful during the decision-making process before prostate biopsy. Over the past few years several new technologies have been developed for the management of PCa. The implementation of robotic surgery allowed an unprecedented refinement of surgical techniques, moreover, the robot-assisted radical prostatectomy procedure is constantly evolving (34). Additionally, artificial intelligence can help physicians to build personalized predictive models, and a recent study demonstrated that with clinical characteristics, their algorithm can improve the prediction of MRI-TRUS fusion targeted biopsy results, which was superior to PSA, its derivates and mpMRI alone (35).

Our study has some limitations. First, this was a single-center retrospective study, and patient selection bias may limit the generalizability. Therefore, the present results may need further validation in prospective multi-center studies with a larger number of patients. second, the PI-RADS v2.1 score was assessed based on T2WI and DWI sequences. However, the PI-RADS performance based on these two sequences was comparable with those studies that have incorporated dynamic contrast-enhanced, which was considered to play a minor role in the diagnosis of PCa. Thirdly, the reference standard was MRI-TRUS fusion targeted biopsy, which may miss potential lesions with a negative MRI but positive pathology.

## Conclusion

Adding PSAD to PI-RADS v2.1 score could significantly improve the diagnostic performance of PCa in PZ. Nevertheless, no substantial improvement in accuracy was observed regarding PCa in TZ.

## Data Availability Statement

The original contributions presented in the study are included in the article/supplementary material. Further inquiries can be directed to the corresponding authors.

## Ethics Statement

The studies involving human participants were reviewed and approved by Jiangsu Vocational College of Medicine. Written informed consent for participation was not required for this study in accordance with the national legislation and the institutional requirements.

## Author Contributions

Guarantor of the article: ZY. Conception and design: WJ and TT. Collection and assembly of data: JY, TT. Data analysis and interpretation: WJ and ZY. All authors contributed to the article and approved the submitted version.

## Conflict of Interest

The authors declare that the research was conducted in the absence of any commercial or financial relationships that could be construed as a potential conflict of interest.

## Publisher’s Note

All claims expressed in this article are solely those of the authors and do not necessarily represent those of their affiliated organizations, or those of the publisher, the editors and the reviewers. Any product that may be evaluated in this article, or claim that may be made by its manufacturer, is not guaranteed or endorsed by the publisher.
